# Insulin autoimmunity associated with vitiligo: a rare case presentation

**DOI:** 10.1530/EDM-22-0369

**Published:** 2024-03-01

**Authors:** Presoon Kuruvilla, Angel John, Ashith Murali

**Affiliations:** 1 Department of Internal Medicine, Caritas Hospital, Kerala, India

**Keywords:** Geriatric, Female, Asian - Indian, India, Skin, Pancreas, Autoimmunity, Neuroendocrinology, Unique/unexpected symptoms or presentations of a disease, March, 2024

## Abstract

**Summary:**

Insulin autoantibody syndrome (IAS) or Hirata’s disease is a rare condition characterized by recurrent fasting hypoglycaemic and postprandial hyperglycaemic episodes. Insulin autoantibodies are diagnostic for the condition. Hirata’s disease has been seen to be associated with other autoimmune conditions. Vitiligo is a common depigmentation disorder whose exact cause is unknown but thought to have an autoimmune aetiology. Although autoimmunity plays a role in the pathogenesis of both the diseases, association between the two has not been reported till date. In our case, a 72-year-old Indian woman with vitiligo for the past 30 years presented with recurrent episodes of fasting hypoglycaemia. She was found to have very high levels of fasting insulin, C-peptide, and insulin antibody and was diagnosed with IAS. Thus, we conclude that the clinical spectrum of Hirata’s disease has to be taken as more heterogenous than previously assumed.

**Learning points:**

- Insulin autoantibody syndrome (IAS) or Hirata’s disease is a rare condition characterized by recurrent fasting hypoglycaemic and postprandial hyperglycaemic episodes in which insulin plays a major role.
- Insulin autoantibodies are diagnostic for IAS. The endocrine insulin and its autoantibodies play a major role in the pathogenesis of the disease.
- Vitiligo is a common depigmentation disorder whose exact cause is unknown but thought to have an autoimmune aetiology.
- IAS and vitiligo are two diseases with autoimmune aetiology which has been seen to be associated with each other (the first case to be reported).
- The clinical spectrum of Hirata’s disease has to be taken as more heterogenous than previously assumed.
- On dealing with autoimmune diseases, we should also keep in mind about other diseases with autoimmune pathogenesis.

## Background

Insulin autoantibody syndrome (IAS) is a rare cause for hypoglycaemia (1). Insulin autoimmune syndrome or autoimmune hypoglycaemia was reported for the first time by Hirata *et al.* in 1970 (1). Symptoms of IAS are recurrent fasting hypoglycaemia, postprandial hyperglycaemia, and post-meal hypoglycaemia which is due to the presence of increased insulin levels and insulin autoantibodies. The disease is mainly reported from Japan making it the third most prevalent cause of hypoglycaemia in the country, but IAS is extremely rare outside Japan (1, 2). Genetically predisposed individuals on exposure to environmental triggers develop insulin antibodies. Mayo Clinic’s diagnostic criteria for endogenous hyperinsulinemic hypoglycaemia are fasting glucose <2.5 mmol/L, insulin concentration >3 U/mL, and C-peptide greater than 3 U/mL. In more than 50% cases of IAS, history of exposure to drugs containing sulfhydryl group has been reported (1). Genetically predisposed individuals on exposure to certain drugs has shown to develop IAS which mainly includes drugs containing sulfhydryl groups and S-S groups – thiamazole, alpha-mercapto propionyl glycine, alpha-lipoic acid, captopril, glutathione, and penicillamine. Non-SH group drugs like steroids and D860 alpha-interferon, albumin have also shown association (1). IAS has also been seen to be associated with other autoimmune diseases, namely, Graves and scleroderma.

Fasting hypoglycaemia with postprandial hyperglycaemia is due to the double-phase mechanism in the pathogenesis of the disease. In the early postprandial phase, the release of Insulin from the beta cells of pancreas results in the formation of Insulin–IAA complexes which hinders the normal physiological action of insulin on glucose that leads to hyperglycaemia. The rising plasma glucose concentration results in the release of excess insulin from the pancreas and leads to increased levels of Insulin–IAA complexes and unbound insulin which binds to its receptor. IAA has high binding capacity but low affinity for insulin which causes spontaneous dissociation of the complexes and release of insulin, which usually occurs 3–4 h post meal leading to mismatch of insulin and glucose in the plasma, consequently hypoglycaemia (1).

Insulin/C-peptide molar ratio rises due to decreased clearance of insulin while C-peptide clearance is unaffected.

Other causes of hypoglycaemia due to abdominal tumours like insulinoma are excluded with magnetic resonance imaging (MRI) imaging.

IAS is generally a self-limiting disease which resolves over months.

Treatment of IAS consists of various therapies which include steroids, azathioprine, and plasmapheresis/mycophenolate mofetil (MMF)/rituximab/cyclophosphamide.

Vitiligo is an acquired depigmentation disorder with increased risk of other autoimmune diseases namely thyroid abnormalities, diabetes mellitus, adrenal insufficiency, and pernicious anaemia (2). It affects approximately 0.5–2% of the population worldwide. It is characterized by depigmented macules and patches on the skin. Vitiligo is associated with various organ-specific antibodies like antithyroid globulins, antithyroid peroxidase, antiparietal cell, and antinuclear antibodies (3).

Since Hirata’s disease is a rare cause of hypoglycaemia, there are not many reported cases and also an association with another disease with a suspected autoimmune aetiology, namely, vitiligo has not been in literature so far.

## Case presentation

A 72-year-old Indian woman presented with multiple episodes of early morning dizziness, anxiety, and confusion. She had similar symptoms about 4 h after meals and were reversed by taking sugary drinks. She had vitiligo for the past 30 years which started from the perioral area. She was also diagnosed with hypertension 1 year back for which she is on Amlodipine once daily. She is not diabetic and has had no exposure to exogenous insulin or oral antidiabetic medication. She denies addictions or habituations, any substance use, or other drugs. She does light household work routinely. She does not have a thyroid swelling.

## Investigations

On evaluation, her weight was 48 kg and height was 150 cm. She was found to have fasting hypoglycaemia (45–50 mg/dL) and postprandial hyperglycaemia (250–300 mg/dL). Fasting insulin levels were found to be >1000 µU/mL (reference range: 2.6–24.9 µU/mL) and C-peptide was 24.87 ng/mL (reference range: 1.1–4.4 ng/mL) measured by electrochemiluminescence assay. To confirm the diagnosis, insulin antibody levels were measured and was observed to be positive (>100.0) (reference range: negative: <10, positive: ≥10). The molar ratio of insulin to C peptide was found to be more than 1. Total cholesterol levels was 144 mg/dL. Other lab tests were within normal limits (complete blood count, liver function test, thyroid function test, renal function test, and serum cortisol). Ultrasonography (USG) and MRI abdomen showed no abnormality of pancreas or any suspicious lesions. Other autoimmune disorders were excluded including systemic lupus erythematosus.

## Treatment

She was treated with steroids (Wysolone 20 mg 1–0–0) and her hypoglycaemia got corrected, her post-prandial hyperglycaemia was controlled with small frequent meals and Voglibose.

## Outcome and follow-up

One year following diagnosis, the hypoglycaemic episodes are controlled by small frequent meals. The steroids were tapered over a period of 20 weeks. Voglibose was given for a period of 6 months and stopped. The patient has not had any hypoglycaemic episodes till date after the proper dietary modifications.

## Discussion

IAS is a rare cause of hyper insulinemic hypoglycaemia with an estimated prevalence of 0.017 cases per 100 000 population worldwide (1). The exact prevalence of the disease is difficult to calculate due to the rarity of the disease, unawareness, and self-limiting nature. Our patient presented with symptomatic fasting hypoglycaemia, and on evaluation, she was found to have high levels of fasting insulin and C-peptide levels. She also had high insulin levels during the episodes of hypoglycaemia. Our patient had a very high titre of fasting insulin >1000, which is very rare in other conditions of endogenous hyperinsulinemia. She had fasting hypoglycaemia, post-prandial hyperglycaemia, and post-meal hypoglycaemia which is characteristically seen with IAA. This finding supports the proposed mechanism in IAS – the high binding capacity of insulin to antibody after meal and its slow release post meal, which causes a mismatch of insulin to glucose in the plasma leading to post-meal hypoglycaemia. The presence of insulin antibodies maybe found in patients receiving insulin but they rarely produce hypoglycaemia or hyperglycaemia as the antibodies seen in this condition have low binding capacity but high affinity to insulin. The endogenous nature of the hyperinsulinemia can be confirmed with the presence of increased C-peptide levels. Although elevated levels of C-peptide can be seen in other causes of hypoglycaemia like sulfonyl urea ingestion, this can be excluded by estimating blood levels of those drugs. Insulin to C-peptide ratio can be taken up as a diagnostic tool for the diagnosis of IAA, although a difference in opinion has been seen. If the molar ratio is more than 1, possibility of IAS can be considered particularly in the presence of IAA. Symptomatic hypoglycaemia with no exposure to exogenous insulin and high titres of insulin autoantibody is highly suggestive of IAS, which was found in our patient.

Imaging studies are usually non-contributory to the diagnosis of IAS. MRI imaging of the abdomen was done, which did not reveal any pancreatic or other intraabdominal abnormalities. Oral glucose tolerance test (OGTT) (Table 1) is usually not essential in the diagnosis of IAS since it might show varying results depending upon the level of insulin. We did an OGTT which showed post-glucose hyperglycaemia followed by hypoglycaemia around 5 h post administration which corresponds with the post-meal hypoglycaemia in our patient. In the presence of two autoimmune conditions namely vitiligo and IAS, we did blood tests to exclude other autoimmune conditions like SLE, which were negative. She had normal thyroid function test. In our opinion, very high titres of insulin with insulin C-peptide ratio more than one in the presence of IAA is diagnostic of IAS.

**Table 1:** Extended oral glucose tolerance test.

Time (h)	Plasma glucose (mg/dL)
0	302
1	371
2	372
3	264
4	178
5	32

There is no definite recommendation for the treatment of IAS due to the rarity of the disease and lack of study. In a drug-related IAS, withdrawal of the offending drug might be beneficial. Considering the autoimmune nature of disease, corticosteroids have shown good results. Dietary modification with small frequent low carbohydrate meals can reduce the post-prandial hyperglycaemia. Along with alpha glucosidase inhibitors, these produce significant control of the post-prandial hyperglycaemia. Our patient was managed with small frequent meals, voglibose, and prednisolone. Steroids were later tapered and she had significant improvement. We are also of the opinion that corticosteroids give good response in IAS. Other therapies like azathioprine and rituximab are suggested for steroid unresponsive cases (1).

Vitiligo is the most common cause of skin depigmentation with a prevalence of 0.1–2% in both adults and children (4). The exact aetiology of vitiligo is not known. One of the proposed mechanisms is the autoimmune cause. Vitiligo patients were found to have autoantibodies in their sera. There are no reports showing insulin autoantibodies in a case of vitiligo. The presence of autoantibodies in this patient with vitiligo adds support to the autoimmune aetiology of the disease. In our patient, the vitiligo was active during the Hirata disease diagnosis. She had generalized vitiligo involving face, trunk, and extremities. Although IAS and vitiligo are two separate disease conditions with autoimmune aetiologies, the co-occurrence of the two makes it a rare case which has not yet been documented. The mechanisms for the co-occurrence are still elusive. This case suggests that the clinical spectrum of IAS is to be taken as more heterogenous than previously assumed.

## Declaration of interest

The authors declare that there is no conflict of interest that could be perceived as prejudicing the impartiality of the study reported.

## Funding

This work did not receive any specific grant from any funding agency in the public, commercial, or not-for-profit sector.

## Author contribution statement

PK treated the patient, researched the data, and reviewed the manuscript. AJ cared for the patient, researched the data, and wrote the manuscript. AM researched the data and contributed to discussion. PK is the guarantor of this work and, as such, had full access to all the data in the study and takes responsibility for the integrity of the data and the accuracy of the data analysis.

## Patient consent

Written informed consent for publication of their clinical details was obtained from the patient.
